# Psychometric assessment of the family satisfaction in the intensive care unit (fs-icu-24) questionnaire among family members of patients admitted to adult general icus in the united kingdom

**DOI:** 10.1186/2197-425X-3-S1-A152

**Published:** 2015-10-01

**Authors:** DA Harrison, P Ferrando-Vivas, SE Wright, E McColl, KM Rowan, FREE Study Investigators

**Affiliations:** Intensive Care National Audit & Research Centre, Clinical Trials Unit, London, United Kingdom; Freeman Hospital, Newcastle upon Tyne, United Kingdom; Newcastle University, Newcastle Clinical Trials Unit, Newcastle upon Tyne, United Kingdom

## Introduction

A number of tools have been developed to seek the views of family members of critically ill patients. The most widely validated is the Family Satisfaction in the Intensive Care Unit 24-item questionnaire (FS-ICU-24).

## Objectives

To establish the psychometric properties of the FS-ICU-24 in the United Kingdom.

## Methods

The Family Reported Experiences Evaluation (FREE) Study recruited family members of patients staying at least 24 hours in 20 participating adult general ICUs between 28 May 2013 and 30 June 2014. Consenting family members were sent a postal questionnaire three weeks after the patient died or was discharged from ICU. Up to four family members were recruited per patient. Only one family member per patient was included in the psychometric assessment, prioritising responses from the patient's next-of-kin and family members with a closer relationship to the patient. Responses were evaluated for high item non-response, floor/ceiling effects, redundancy and items measuring a construct other than intended. Internal consistency was evaluated with item to own scale correlations and Cronbach's alpha. Confirmatory factor analysis was used to test the goodness of fit of the two-factor solution (*satisfaction with care* and *satisfaction with decision-making*) from the North American validation study. Exploratory factor analysis was undertaken to further explore the underlying structure.

## Results

12,346 family members of 6380 patients were recruited to the FREE Study and 7173 (58%) family members of 4615 patients returned a completed questionnaire. The 4615 included family members (one per patient) had a mean age of 56 years; 67% were female; 46% were the patient's partner, 26% a child, 10% a parent and 18% other relationships. Six items (relating to symptom management and the decision-making process) had >10% non-response (predominantly 'N/A'); these were not considered to represent lack of comprehensibility or acceptability. The item 'concern and caring by ICU staff' had a possible ceiling effect (72% 'Excellent'). Eleven items were flagged for redundancy; all were just over the threshold (item to own scale correlation > 0.8) and captured different aspects of satisfaction. Internal consistency was high (Cronbach's alpha 0.96 overall, 0.94 for *satisfaction with care* and 0.93 for *satisfaction with decision-making*). The two-factor solution was not a good fit (root mean squared error of approximation 0.095). Exploratory factor analysis indicated that *satisfaction with decision-making* encompassed two constructs—*satisfaction with information* (first six items) and *satisfaction with the decision-making process* (final four items). Coding of free text responses identified some discordance between scores and comments.

## Conclusions

While the FS-ICU-24 has adequate psychometric properties, initial analysis of free text responses suggests scope for further improvement.

## Grant Acknowledgment

NIHR Health Services and Delivery Research Programme.

